# The Open-Air Treatment of Tuberculosis

**Published:** 1899-11-18

**Authors:** 


### THE OPEN-AIR TREATMENT OF TUBERCULOSIS.

THE discussion on the open-air treatment of tuberculosis which was commenced on Tuesday at the Royal Medical and Chirurgical Society cannot be said to have carried the matter very much further. But it was made clear that in many sanatoria which claim good successes the amount of open air obtained is a very variable quantity, and is supplied in ways which are not always of the best. Professor Clifford Allbutt was especially sarcastic on the subject of draughts. The treatment of tuberculosis by open air he approved of, but its treatment by draughts was another matter. He could but describe the rooms in some of the sanatoria which he had visited as "careering-grounds for all the winds of heaven," and he was quite sure that such exposure to cold as was sometimes endured had a distinctly depressing effect. Patients might after a time become indifferent to it, but the cyanosis which some of them exhibited seemed to him to be a sign of enfeebled circulation. Nor could he agree with all that was held by some physicians in regard to the necessity for isolation. A certain amount of interest in current affairs—even a certain amount of excitement—notwithstanding that it might cause a slight elevation of temperature, by raising the spirits raised the general vitality, and he strongly protested against depriving patients of companionship, and even of interesting books—a system which he characterised as one of enforced imbecility, a couple of months of which he was sure would send him off his head.

Again, he urged caution in regard to many of the reported cases of cure. "We are all," he said, "week by week, seeing alleged cures coming back by no means cured"; and he insisted on the fact, too often forgotten, that the presence of physical signs was an indication of comparatively advanced disease, and that their removal, while it meant improvement, could not in any proper sense be taken as a proof of cure. The fact is that much that we hear about cures in three months is all nonsense, and no doubt Professor Allbutt's criterion is a much more real one. "Have they," he would ask, "been at their old work amid their old conditions for a twelvemonth without a recurrence?" If so, one may begin to speak of cure.

There can be little doubt that the boom in sanatoria with which we are threatened has certain drawbacks. In opening the discussion, Dr. Kingston Fowler pointed out many defects in foreign sanatoria, some of which are, indeed, mere hotels, in which the patients do as they like, feed as they like, and shut or open their windows as they like. He caused some amusement by mentioning a sort of "strike" which had occurred at one establishment, the patients having signed a round robin to the effect that if certain windows were not shut they would not come down to breakfast. Terrible threat! Of the advantages, however, of sanatorium treatment when properly managed he was strongly convinced. The great hotel with a physician attached calling itself a sanatorium is another affair. What is to be insisted on is that the sanatorium treatment is not a matter of mere exposure to open air, or of taking so many hours of "open air" as if it were a medicine, and then passing the evening in a hot recreation room. It is a system of proper and somewhat full feeding; of life in the open air as far as may be, and in clean air when within doors; of graduated exercise according to the strength; and more especially of careful and constant medical supervision, so that each day's exercise and food and exposure may be attuned to the condition of the patient. In details the various sanatoria differ greatly, but, as Dr. Kingston Fowler put it, "the system which aims at increasing the resisting power of the body is the one which will be most likely to succeed." In such a system open air is the central fact, but it is far from being everything.

